# Assessment of the Burden of Multiple Sclerosis Patients' Caregivers in Saudi Arabia

**DOI:** 10.7759/cureus.6658

**Published:** 2020-01-14

**Authors:** Hussein Algahtani, Bader Shirah, Abdulrahman Bayazeed, Abdullah Alghamdi, Malik Almailabi, Mohammed Algharib, Faisal Alkahtani

**Affiliations:** 1 Neurology, King Abdulaziz Medical City, Jeddah, SAU; 2 Neurology, King Saud Bin Abdulaziz University for Health Sciences, Jeddah, SAU

**Keywords:** multiple sclerosis, burden, caregivers, saudi arabia

## Abstract

Introduction

Multiple sclerosis (MS) is a disease that constitutes a high burden on both patients and caregivers. Although many studies have assessed the burden of MS caregivers worldwide, no such studies have been conducted in Saudi Arabia. In this study, we aim to assess the burden of MS patients' caregivers in Saudi Arabia.

Methods

This cross-sectional study included caregivers of MS patients (for >1 year) who visited the neurology clinic at King Abdulaziz Medical City, Jeddah, Saudi Arabia, between July 2017 and December 2018. The study utilized the Zarit Burden Interview (ZBI) to assess the burden on MS patients' caregivers. In addition, the caregivers' demographic profile and certain information regarding the patient were also collected through an oral interview.

Results

There were a total of 219 respondents, of which 117 (53.4%) male caregivers. For ZBI, the majority of caregivers reported little or no burden (57.1%), followed by a mild to moderate burden (30.1%), then a moderate to severe burden (10.5%), and only five (2.3%) reported a severe burden.

Conclusion

Our results showed a limited burden of MS on the life caregivers of MS patients. We stress the importance of assessing the burden in MS patients and caregivers as routine practice with the other important measures such as quality of life and medication compliance. The finding of this study will help in encouraging medical centers to establish more specialized MS clinics that put into consideration the psychological factors, burden of the disease, multidisciplinary approach, and support groups, which are currently few in number.

## Introduction

Multiple sclerosis (MS) is a chronic inflammatory disease of the central nervous system (CNS) characterized by demyelination, axonal loss, and neurodegeneration [1]. The relapsing-remitting form of the disease (85% of cases) initially starts with sensory and motor deficits, coordination problems, and bladder control dysfunction. These symptoms are followed by permanent neurological deficits, progressive disability, and medical and physical deterioration over the following two to three decades [2]. The etiology of this disease is unknown. Some studies suggest that both genetic and environmental factors play a causative role [3]. According to the World Health Organization (Atlas of MS in 2013), there were 2.3 million MS-affected people worldwide with variable prevalence ranging from 15/100,000 to 250/100,000 population. The number is likely to be much higher as many people with MS are undiagnosed [4]. The Middle East has a low prevalence of MS when compared to North America and Europe. However, according to a paper that was published in 2013 and based on a panel of experts in the Gulf region regarding the epidemiology of MS suggest an increase in the number of cases [5].

Clinically, patients with MS experience recurrent relapses, presenting with neurological impairment involving multiple systems, including sensory, motor, coordination, and bowel and bladder. The disease becomes chronic and progressive with time with the subsequent development of motor disability and cognitive impairment [6]. The quality of life of patients with MS and their caregivers is affected by the alteration of the level of independence, social interaction, and psychological status [7]. There are several studies that assessed the quality of life of MS patients in Saudi Arabia [8-9]. In addition, MS has a high burden on caregivers [10]. Multiple factors have been found to affect the degree of caregiver burden, including the severity of the disease, type and severity of symptoms, caregiver age and health, presence of social support, and the number of hours spent assisting the patient. These factors can expose the caregivers to high levels of stress affecting mental and physical health, quality of life, and social responsibilities [11].

Although there are many studies that assessed the quality of life of MS caregivers and the burden of the disease on them around the world, no studies have been conducted in Saudi Arabia. Most of these studies have been published from Europe and North America where the disease prevalence and severity are high [12]. We believe that MS in Saudi Arabia has cultural, social, and economic aspects that are distinct from the other countries in the region and worldwide. These include relationships among family members, free medical care, governmental financial benefits, and social stigma. For these reasons, we are conducting this study to assess the burden of MS patients’ caregivers in our population and compare the results with other studies in the region and worldwide.

## Materials and methods

This study is a cross-sectional survey conducted at King Abdulaziz Medical City, Jeddah, Saudi Arabia. The medical city is one of the largest hospitals in the western region of Saudi Arabia, and it receives cases from all social and economic classes, including non- Saudis; thus, the demographics of the hospital clinic population compare with the general population of Saudi Arabia. Caregivers for patients with MS who visited the neurology clinic from July 2017 to December 2018 were included. Caregivers who cared for their patients for less than one year were excluded. The sample size was calculated by using the Raosoft® software (Raosoft Inc, Seattle, WA). The total number of the MS patient population at the neurology department is 400 patients. The required minimum sample size was determined to be 197; the final sample size will be taken as 217 to account for a 10% non-response rate.

The study utilized the Zarit Burden Interview (ZBI) to assess the burden on MS patients’ caregivers. The ZBI was developed to measure subjective burden among caregivers of adults with dementia. Items were generated based on clinical experience with caregivers and prior studies resulting in a 22-item self-report inventory that examines the burden associated with functional or behavioral impairments and the home care situation. The items are worded subjectively, focusing on the affective response of the caregiver. Each question is scored on a five-point Likert scale, ranging from never to nearly always present. Total scores range from 0 to 88. The cut-off values were 0-21 little or no burden, 21-40 mild to moderate burden, 41-60 moderate to severe burden, and 61-88 severe burden. In addition to ZBI, the demographic profile of the caregivers was collected. We also collected certain information in regard to the patient through an oral interview, which includes the following (gender, relationship, degree, job, city, duration of caring, and average of daily hours of caring and income).

All numerical values and demographic data obtained from the questionnaire were calculated and presented as frequency and percentage. Descriptive statistics were used including means, median, and standard deviation. All collected data were entered and analyzed through the Statistical Package for the Social Sciences (SPSS) version 23 (IBM Corp., Armonk, NY). All data collection sheets were anonymous and confidentiality of information was maintained. This study is conducted under the permission of the ethics committee at King Abdullah International Medical Research Center.

## Results

**Figure 1:**
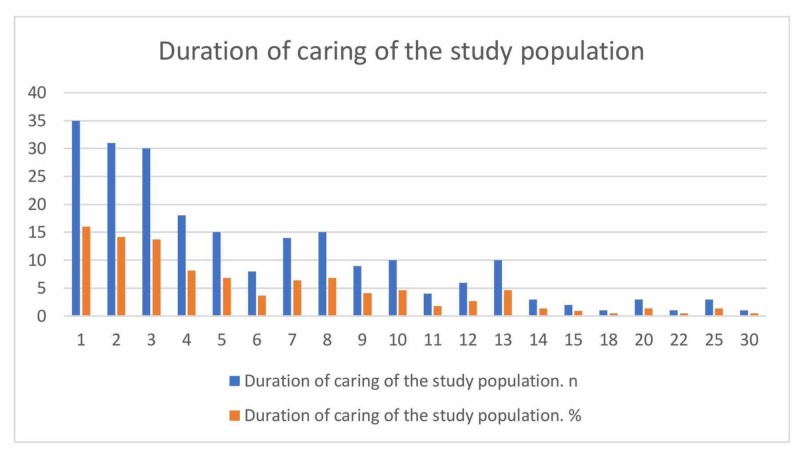
Duration of caring of the study population

**Figure 2:**
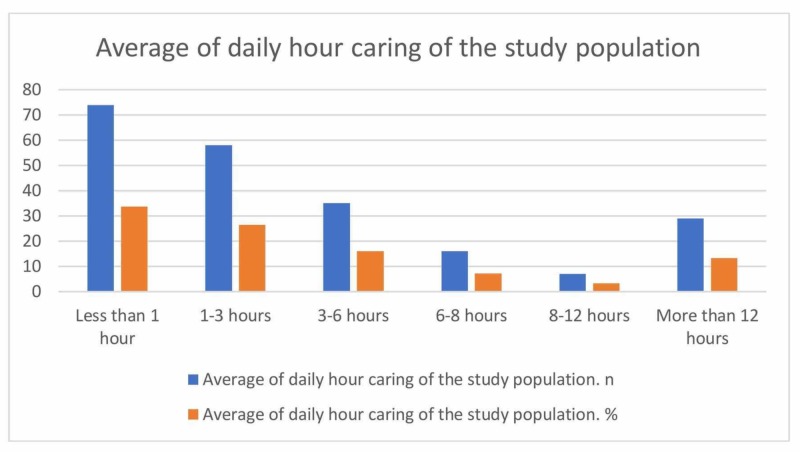
Average of daily hours of caring of the study population

**Figure 3:**
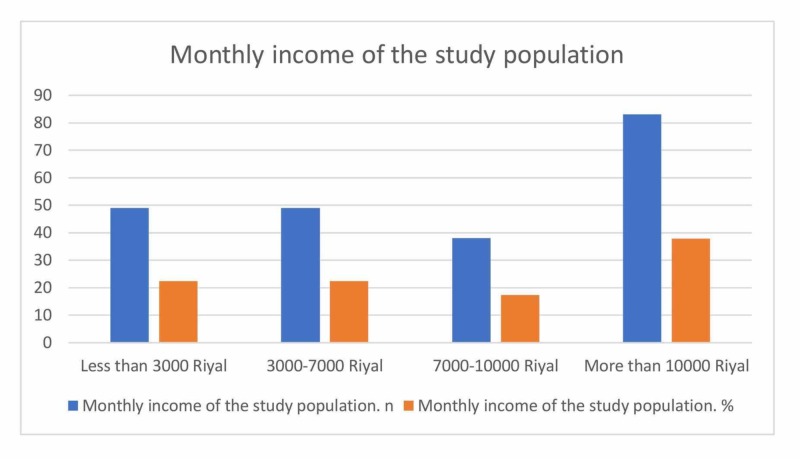
Monthly income of the study population

A total of 219 questionnaires were collected. The questionnaire included two sections; the first section was for caregivers. The number of male caregivers was 117 (53.4%) while the number of female caregivers was 102 (46.6%). In terms of relationship with the patient, most of the caregivers were a spouse (33.9%) followed by a sibling (22%), a parent (21.6%), a son/daughter (14.7%), and a second-degree relative (7.8%). Regarding caregiver degree, the majority were bachelor degree holders (65.8%) while only three (1.4%) were illiterate. Moreover, 140 (63.9%) were employed and 79 (36.1%) were unemployed. Caregivers and their patients were living in three different cities in the Western region of Saudi Arabia (Jeddah (52.5%), Makkah (16.4%), and Taif (9.1%)). Eight caregivers (3.7%) were taking care of patients for a period of more than 20 years (Table 1, Figure 1). Twenty-nine caregivers (13.2%) were spending more than 12 hours caring for their patients (Table 2, Figure 2). Around 40% of caregivers were earning more than 10000 Saudi Riyal per month (Table 3, Figure 3).

**Table 1:** Duration of caring of the study population

Table: Duration of caring of the study population		
Duration of caring (years)	n	%
1	35	16
2	31	14.2
3	30	13.7
4	18	8.2
5	15	6.8
6	8	3.7
7	14	6.4
8	15	6.8
9	9	4.1
10	10	4.6
11	4	1.8
12	6	2.7
13	10	4.6
14	3	1.4
15	2	0.9
18	1	0.5
20	3	1.4
22	1	0.5
25	3	1.4
30	1	0.5
Total	219	100

**Table 2:** Average of daily hours of caring of the study population

Table: Average of daily hours of caring of the study population		
Average of daily hour caring	n	%
Less than 1 hour	74	33.8
1-3 hours	58	26.5
3-6 hours	35	16
6-8 hours	16	7.3
8-12 hours	7	3.2
More than 12 hours	29	13.2
Total	219	100

**Table 3:** Monthly income of the study population

Table: Monthly income of the study population		
Income	N	%
Less than 3000 Riyal	49	22.4
3000-7000 Riyal	49	22.4
7000-10000 Riyal	38	17.4
More than 10000 Riyal	83	37.9
Total	219	100

Caregivers who participated in our study were taking care of 65 (29.7%) male patients while female patients numbered 154 (70.3%). Regarding the patient's degree, the majority were holders of a Bachelor's degree (58%) while only six (2.7%) were illiterate. Moreover, 85 (38.8%) were employed and 134 (61.2%) were unemployed.

For ZBI, the majority of caregivers reported little or no burden (57.1%), followed by mild to moderate burden (30.1%), then moderate to severe burden (10.5%), and only five (2.3%) reported severe burden (Table 4).

**Table 4:** Zarit Burden Interview responses of the study population

Table: Zarit Burden Interview responses of the study population						
	Item	0. Never	1. Rarely	2. Sometimes	3. Quite Frequently	4. Nearly Always
1	Do you feel that your relative asks for more help than he/she needs?	31.50%	24.48%	28.76%	5.93%	7.30%
2	Do you feel that because of the time you spend with your relative, you don’t have enough time for yourself?	48.85%	16.43%	25.57%	4.10%	5.02%
3	Do you feel stressed between caring for your relative and trying to meet other responsibilities for your family or work?	47.03%	13.69%	23.74%	9.13%	6.39%
4	Do you feel embarrassed about your relative’s behavior?	68.03%	11.87%	10.04%	7.30%	2.73%
5	Do you feel angry when you are around your relative?	68.03%	15.52%	11.41%	3.65%	1.36%
6	Do you feel that your relative currently affects your relationship with other family members or friends in a negative way?	86.66%	14.61%	12.32%	5.02%	1.36%
7	Are you afraid of what the future holds for your relative?	25.11%	8.84%	25.11%	19.63%	23.28%
8	Do you feel your relative is dependent upon you?	25.11%	15.98%	27.39%	18.26%	13.24%
9	Do you feel strained when you are around your relative?	79.90%	8.67%	8.21%	2.28%	0.91%
10	Do you feel your health has suffered because of your involvement with your relative?	68.49%	12.78%	12.78%	4.10%	1.82%
11	Do you feel that you don’t have as much privacy as you would like, because of your relative?	75.34%	10.04%	10.95%	2.73%	0.91%
12	Do you feel that your social life has suffered because you are caring for your relative?	65.75%	19.17%	9.58%	3.19%	2.28%
13	Do you feel uncomfortable about having friends over, because of your relative?	79.90%	10.50%	5.93%	2.73%	0.91%
14	Do you feel that your relative seems to expect you to take care of him/her, as if you were the only one, he/she could depend on?	37.44%	18.77%	17.35%	9.13%	7.35%
15	Do you feel that you don’t have enough money to care for your relative, in addition to the rest of your expenses?	39.26%	13.96%	22.83%	13.69%	10.50%
16	Do you feel that you will be unable to take care of your relative much longer?	77.16%	7.76%	12.32%	1.36%	1.36%
17	Do you feel you have lost control of your life since your relative’s illness?	66.21%	15.06%	11.41%	4.10%	3.19%
18	Do you wish you could just leave the care of your relative to someone else?	81.27%	8.21%	8.21%	1.36%	0.91%
19	Do you feel uncertain about what to do about your relative?	43.83%	18.21%	26.94%	8.21%	2.73%

20	Do you feel you should be doing more for your relative?	16.89%	12.32%	25.11%	23.74%	21.91%
21	Do you feel you could do a better job of caring for your relative?	19.17%	11.87%	32.87%	21.00%	15.06%
22	Overall, how burdened do you feel while caring for your relative?	40.63%	24.65%	17.35%	10.04%	7.30%

## Discussion

In social sciences, quality of life is a term used to broadly encompass a subjective sense of the goodness of multiple aspects of life. In medicine and public health, quality of life refers to those aspects of high functions that are influenced by health status.

Measuring the quality of life in different populations can identify subgroups that are at high risk for mental or physical health complications. This may help establish guidelines, policies, or interventions to improve their health. Caring for patients with chronic diseases, such as MS, may have a substantial impact on the mental, physical, and social aspects of caregivers. This has been recognized by policymakers in most countries worldwide. A treatment that improves the caregivers’ quality of life is a definite benefit to be considered in a cost-utility analysis. Unfortunately, in a consensus statement by 20 key opinion leaders, neurologists and MS experts representing various countries of the Arabian Gulf have not discussed or mentioned any data on the quality of life of patients with MS or their caregivers [13]. On the other hand, the caregiver burden has been defined as the degree or type of stress or strain that caregivers experience and the challenges they face as a result of caring tasks or restrictions that cause discomfort for them as caregivers. It is a multidimensional response to several stressors associated with the caregiving experience. These include social, psychological, physical, emotional, and financial stressors.

An assessment of the caregiver burden is necessary to draw conclusions about the effectiveness of family interventions and to judge the situation of the caregiver. It is important to assess the caregiver burden to develop the necessary interventions for reducing this burden and examining their effectiveness [14].

The quality of life of caregivers is influenced by a variety of factors other than the patient’s health status. These factors include the caregiver’s gender, relationship with the patient, level of education, duration of caring, average daily hours of caring, and income. Nowadays, there is an increasing trend toward the evaluation of the quality of life for MS patients and their caregivers. In literature, we found multiple studies that assessed the quality of life in MS patients’ caregivers, which showed a decrease in the quality of life as compared to the general population [11]. This was also reflected by our study in which around 42% of the participants reported mild, moderate, or severe burden.

Studies conducted on different neurological diseases have shown that caregiving is associated with increased distress, both emotionally and socially. This can lead to physical and psychological distress to caregivers with subsequent institutional placement and deterioration or worsening of the course of the disease [15]. Unfortunately, this was not studied in depth in our study, which is considered a limitation.

In 1980, Zarit et al. developed a 29-item questionnaire that aimed to assess the level of burden experienced by caregivers of demented and disabled persons. It is widely used, and the current version is composed of 22 items [16]. The Chronbach α for this instrument ranges from 0.85 to 0.94 [17]. In this study, we chose this questionnaire because of its simple format, easy to understand and fill by the participants, and it takes a few minutes to be filled up. Although it is a very simple questionnaire, its design does not negatively affect its usefulness and reliability. There are other questionnaires that are available in the Arabic language such as the Multiple Sclerosis International Quality of Life (MusiQoL) and the 36-Item Short Form Survey (SF-36).

In our MS patients’ caregivers, there is an apparent propensity toward a decrease in the mean results with the duration of care and the age of both patients and their caregivers. These observations can be attributed to the increased burden due to the stress of care on the caregivers and the progressive disability of the patients throughout the course of the disease, with more symptoms of MS or other comorbidities that occur with age. Older patients with a progressive and long-term disease appraise their quality of life at a much lower level as compared to younger patients. This indicates that the burden of caregivers is mainly influenced by the age and duration of care [18].

Around 50% of MS patients are unemployed 10 years after diagnosis. Factors leading to unemployment include increasing disease severity, increasing age, and functional changes [19]. In our study, 61.2% were unemployed. In a meta-analysis published by Dorstyn et al. [20], 33 cross-sectional studies were reviewed with employment participation ranged from 12% to 74% with an average of 44% across all studies. This indicates that our unemployment rate is higher than that reported by most of the European and North American nations.

Familism is a term used to describe the development of a feeling of duty among the members of the family group and the orientation towards the welfare of one’s own family. It refers to the core values of a family type with emphasis on the well-being of the family group against the interest of each one of its members [20]. This was reflected in our study since most of the caregivers were immediate family members, including a spouse (33.9%), a sibling (22%), a parent (21.6%), a son/daughter (14.7%), and a second-degree relative (7.8%). Family social support in much more prominent in Asian countries as compared to European countries. Caregiving in Asia is associated with familism rather than individualism, which was demonstrated in several studies [21].

In our study, caregiving was provided by males in 53.4% of the study population. Although it is traditionally known that caregiving is a responsibility of the woman, and men are considered strangers to the caregiving responsibilities, this was not observed in our study. This can be explained by the fact that MS is a disease that affects more females (70.3%) with their spouses being the caregivers, and if a male is affected, the caregiver will be a male rather than a female.

In our study, it was determined that 58.9% of caregivers provided the patients with care for one to five years while 8% provided the care for more than 20 years. The time spent for the care was three hours or less in 60.3% while in 13.2% of the study population, the care was more than 12 hours. Our results are similar to previously published studies [22].

In several studies conducted on the burden of MS on patients’ caregivers [23], employed caregivers have higher mean scores as compared to those unemployed. This might be due to the fact that employment caused stress on caregivers with subsequent depression, exhaustion, and an increase in health problems such as insomnia, diabetes, and arthritis. This was reflected in our studies with the mean scores received by the employed patient's caregiver were found to be higher compared to those who are unemployed. These results are similar to those published by Zarit et al., Ozmin et al., and others [16,24].

Although the monthly income of more than half of the sample size was more than 10,000 SR, more than half of the respondents admit that financial problems are major issues when taking care of patients with MS. Healthcare is provided for free for all citizens in Saudi Arabia. This conflicting result may be due to a lack of proper home health care support and difficulties with providing the physical care requirements other than medications. Specialized MS clinics with social workers being part of the team may solve such problems.

Among the caregivers included in our study, spouses represented around one-third of the sample size with higher burden mean scores as compared to other relatives. This result is similar to the study conducted by Figvid et al. [25] who found that the spouses providing care to MS patients experienced more distress with lower life qualities as compared to other groups.

The main limitation of our study is that it was cross-sectional, involving subjects from only one center. Therefore, our subjects may not be representative of the general population of caregivers of MS patients in Saudi Arabia. In addition, the perception of patients regarding the quality of care given in the family was not assessed. It is well- known that age affects the care burden, and younger caregivers experience a heavier burden as compared to older caregivers. Unfortunately, the mean scores received by the participating caregivers of different age groups were not assessed. The strength of our study is that this is the first study assessing the burden of MS on caregivers in Saudi Arabia. This is considered an urgent call to conduct more studies on this subject.

## Conclusions

In conclusion, our results showed a limited burden of MS on the life caregivers of MS patients. Familism is a possible explanation of the little or no burden results in more than half of the participants in our study. We stress the importance of assessing the burden in MS patients and caregivers as a routine practice with other important measures such as quality of life and medication compliance. The finding of this study will help in encouraging medical centers to establish more specialized MS clinics that put into consideration the psychological factors, burden of the disease, multidisciplinary approach, and support groups, which are currently few in number.
